# Seismic responses of rectangular subway tunnels in a clayey ground

**DOI:** 10.1371/journal.pone.0204672

**Published:** 2018-10-17

**Authors:** Lei Zhang, Yong Liu

**Affiliations:** 1 School of Civil Engineering and Architecture, Wuhan University of Technology, Wuhan, P.R. China; 2 State Key Laboratory of Water Resources and Hydropower Engineering Science, Institute of Engineering Risk and Disaster Prevention, Wuhan University, Wuhan, P. R. China; Technische Universiteit Delft, NETHERLANDS

## Abstract

Observations from past earthquakes have shown that subway tunnels can suffer severe damage or excessive deformation due to seismic shaking. This study presents the results of finite element analyses on subway tunnels installed in normally consolidated clay deposits subjected to far-field ground motions. The clay strata were modelled by a hyperbolic-hysteretic constitutive model. The influences of three factors on the seismic response of the clay-tunnel systems were examined, namely ground motion intensity, tunnel wall thickness and clay stiffness. Furthermore, the computed racking deformations of the tunnel were compared with several analytical estimations from the literature, and the relationship between racking ratio and flexibility ratio for rectangular tunnels installed in normally consolidated clay deposits was proposed. The findings may provide a useful reference for practical seismic design of tunnels.

## Introduction

To ensure sustainable development, much attention has been paid to constructing more tunnels and other underground structures in view of the relatively limited land resources and the increasing number of immigrants encountered in most of metropolitan cities. The performance of these tunnels and underground structures against natural hazards (e.g. earthquakes) deserves to be examined fully. Many previous publications have reported that tunnels and underground structures can suffer severe damage or excessive deformation due to seismic shaking [1–4]. To date, the guidelines for seismic design of tunnels predominantly rely on simplified methods [3,5–13]. At the absence of the dynamic soil-structure interaction mechanism, those methods may result in a considerable overestimation or underestimation of the seismic response of a tunnel [14–15].

Numerous studies have been performed to investigate the seismic response of tunnels considering the dynamic soil-structure interaction, using a 1-g shaking table or centrifuge tests [14,16–26] and numerical analyses [4,14–15,27–38]. Most of these studies mainly focused on the tunnels installed in dry sands or liquefiable soils. Only a few exceptions can be found. For instance, Amorosi and Boldini [30] performed 2D FE simulations to investigate the influences of soil constitutive models (namely visco-elastic and visco-elasto-plasic effective stress models) on the seismic response of a circular tunnel embedded in clayey soils; Moss and Crosariol [22] conducted 1-g shaking table tests to examine the seismic behaviour of rectangular tunnels installed in soft clay deposits, focusing on responses of the ground surface acceleration and lateral distortion of the tunnel structure; Chen et al. [24] carried out both 1-g shaking table tests and 3D FE analyses to investigate the damage mechanism of a 3-storey and 3-span subway structure installed in soft clays subjected to strong ground motions, with the focuses placed on the acceleration and lateral displacement responses of the subway structure. In general, compared to a sand deposit, the clay deposit’s stiffness is comparatively smaller and the hysteresis damping effect is more significant. Besides, when subjected to strong ground motions, the excess pore pressures generated within clay deposits may remain at a relatively low level, and liquefaction of the clay deposits is not likely to happen; this seismic behaviour of the clay deposit is significantly different from that of a saturated sandy deposit. For these reasons, the clay deposits will be considered in this study.

In coastal cities such as Singapore and Shanghai, where large portions of the land are underlain by thick layers of normally consolidated clays, many superstructures and underground structures are built on/in normally consolidated clay deposits. A distinct feature of normally consolidated clay is its significant amplification effect on earthquake-induced ground motions for small to moderate ground motion intensities [39–43]. As a result of this amplification, the structures built on or installed in this type of clay strata may suffer amplified loading arising from the surrounding soils. Furthermore, given that the relevant studies involving underground structures in a normally consolidated clay stratum are still relatively few, especially for those involving far-field ground motions, the seismic interaction mechanism of rectangular tunnel-clay system has yet to be fully understood. Hence, it will be beneficial to carry out such research to advance the understanding of their seismic performance.

In this study, a series of two-dimensional (2D) plane strain finite element (FE) analyses was performed to investigate the seismic response of rectangular tunnels that are installed in normally consolidated clay deposits and subjected to far-field ground motions. A rectangular tunnel is widely encountered in real practice; for instance, the cross-river tunnel (i.e. Hejiangtao Tunnel) in Hengyang city (China) is a rectangular shape with 11.3m in tunnel height and 10.3m in tunnel width. The clay was modelled following a hyperbolic-hysteretic soil model, and the basic properties are listed in Table 1. This hyperbolic-hysteretic model was developed based on results from a series of resonant column and cyclic triaxial tests on kaolin and soft marine clays [43–44], which can favourably well replicate the dynamic behaviour of soft clay under seismic loadings, namely hysteresis damping, strain level- and loading cycle-dependent stiffness reduction, and significant amplification effect on seismic-induced motion. The current study focused on the ground acceleration response and tunnel structure responses, with the influences of ground motion intensity, tunnel wall thickness and clay stiffness taken into consideration; seismic tunnel structure responses comprise the shear force, bending moment and lateral deformation of the left and right tunnel walls during the seismic shaking events.

**Table 1:** Properties of clay adopted in this study.

Property	Value
Poisson’s ratio	0.3
Density (kg/m^3^)	1,600
Effective unit weight (kPa)	6
Frictional angle of clay	25°
Coefficient of earth pressure at rest	0.5774
Small-strain shear modulus (kPa)	*A*(*p*_0_^’^)^0.653^

Note: *p*_0_^’^is the initial mean effective normal stress, with the units of kPa; *A* is the stiffness parameter, with the values shown in Table 3.

## Input far-field ground motions

**Fig 1:**
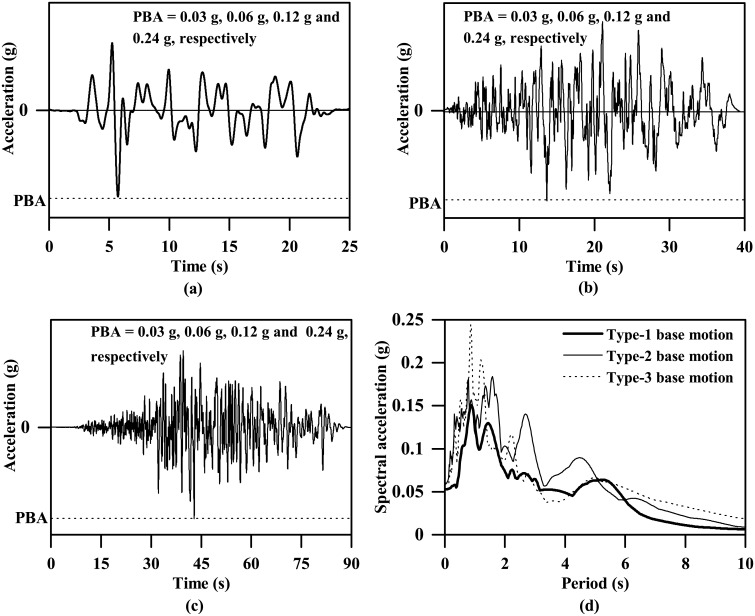
Input base accelerations used in this study. (a) time history for Type-1 base motion, (b) time history for Type-2 base motion, (c) time history for Type-3 base motion, (d) response spectra for the three types of base motions with a peak base acceleration (PBA) of 0.06 g (5% damping).

**Fig 2:**
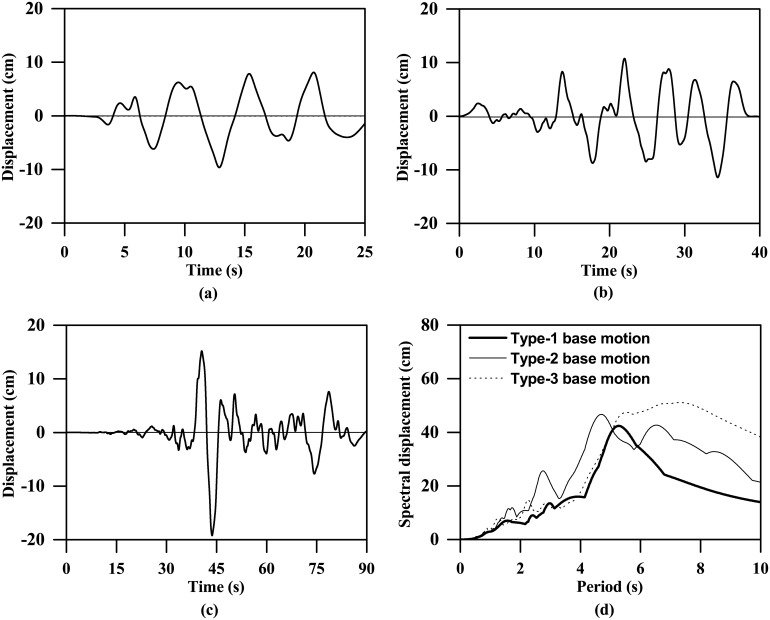
Displacement plots of the input base motions (PBA = 0.06 g). (a) time history for Type-1 base motion, (b) time history for Type-2 base motion, (c) time history for Type-3 base motion, (d) response spectra (5% damping).

As Fig 1 shows, three types of base motions were adopted in this study. The base motion with a peak acceleration of 0.06g shown in Fig 1(a) represents a type of far-field shaking event that may be felt in Singapore. It is a synthesized signal with reference to the past seismic-induced motions measured in Singapore, which was also used by Zhang [45] and Zhang et al. [46] for both the centrifuge tests and FE analyses. In addition, the ground motions shown in Fig 1(b) and 1(c) were compiled based on two horizontal earthquake records (east-west direction) measured at Stations El Monte-Fairview Av and TTN005 during the 1992 Landers and 1999 Chi-Chi earthquakes, respectively. These two earthquake records were selected from the Ground Motion Database provided by the Pacific Earthquake Engineering Research (PEER) Centre. They have site-to-source distances of approximately 136 km and 83 km, respectively, which fall within the scope of far-field ground motion according to Dadashi and Nasserasadi [47] and Bhandari et al. [48]. For simplicity, these ground motions are termed Type-1, Type-2 and Type-3 base motions. The response spectra corresponding to a peak acceleration of 0.06g are plotted in Fig 1(d). The displacement plots of the input base motions are also provided in Fig 2. As can be seen, due to the differences in time duration and frequency content, the peak displacements are generally different even though the peak accelerations are same. In addition, to investigate the influence of ground motion intensity, each of these base motions was scaled into four ground motions with peak values of 0.03g, 0.06g, 0.12g and 0.24g.

## Numerical modelling procedure

The present numerical analysis consists of two phases, namely gravity loading and earthquake loading phases. Initial stress condition is assigned to the soil domain to account for the gravitational stress field of the clay-tunnel system in the gravity loading phase; subsequently, far-field ground motions are excited at the model base in the earthquake loading phase. Some other information on the numerical modelling procedure is given below.

### Basic information

**Fig 3:**
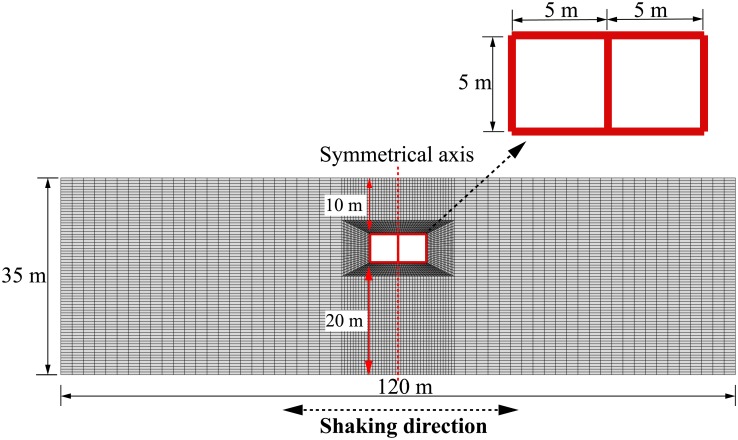
Two-dimensional (2D) finite element model of the clay-tunnel system.

As Fig 3 shows, the 2D plane strain FE model of the clay-tunnel systems employed in this study was set up using ABAQUS, which contains 7900 linear quadrilateral elements and 140 linear beam elements to model the clay and rectangular tunnels, respectively. Although there are many different suggestions (e.g. [49–50]) concerning the element size in seismic numerical analyses, it is generally accepted that the element number per seismic wavelength of 10 is sufficient for accurately modelling the seismic wave propagation. This criterion is strictly followed in the present study (refer to reference [51] for further relevant information). In addition, although not included herein, a pure clay FE model was also employed to obtain the free-field ground acceleration response for comparison purposes. As Fig 3 shows, the rectangular tunnel is divided by a central column into two square parts and embedded at a depth of 10 m below the ground surface, which was adopted to represent a subway station. As Table 2 shows, the central columns were assumed to have a spacing of 8 m and cross-sectional dimensions of 0.4 m × 0.4 m. To obtain a flexural rigidity of the central column equivalent to the flexural rigidity modelled in three-dimensional space, the Young’s modulus of the central column was reduced by 20 times compared to that of the tunnel wall in the FE analyses. The thicknesses of the four sides of the tunnel structure, termed tunnel wall thickness, were kept equal, ranging from 0.2 m to 0.8 m. More information on the mechanical and geometric properties of the tunnel structure is provided in Tables 2 and 3.

**Table 2:** Properties and dimensions of tunnel structure (reinforced concrete).

Property	Value/range
Young’s modulus of tunnel structure (kPa)	30,000,000
Poisson’s ratio of tunnel structure	0.2
Density of tunnel structure (kg/m^3^)	2,500
Nominal cross-section of central column (m)	0.4×0.4
Nominal spacing of central column (m)	8

**Table 3:** Parameters adopted in the numerical parametric studies.

Property	Value
Thickness of tunnel wall (m)	0.2; 0.4; 0.6; 0.8
PBA (g)	0.03; 0.06; 0.12; 0.24
Stiffness parameter *A*	515; 1030; 2060; 4120; 8240

Note: PBA is the peak base acceleration of the input base motion; small-strain shear modulus *G*_max_ = *A*(*p*_0_^’^)^0.653^, where *p*_0_^’^ is the initial mean effective normal stress with the units of kPa.

### Boundary conditions

To minimize the possible boundary reflection effect arising from the truncated lateral boundaries, with the reference to the previous studies by Tsinidis et al. [25], Bao et al. [37] and Tsinidis [36], the lateral boundaries were adopted to have a dimension 12 times the tunnel width. In addition, the nodes at the same depths on the two lateral boundaries were constrained to have the same horizontal and vertical displacements. This approach has been validated to be effective in replicating a free-field ground motion, and was adopted in many previous studies [15,33,42,51]. In addition, a rigid boundary condition was applied at the bottom of the FE model so that the model base simply moves along the direction of the shaking.

### Clay-tunnel interface

Like the studies by Debiasi et al. [33] and Tsinidis et al. [15], hard-contact and penalty friction algorithms were used to model the normal and tangential behaviours of the soil-tunnel interface, respectively. For tangential behaviour, the friction coefficient was set equal to 0.4663, which was obtained by assuming a frictional angle the same as that of clay. The effect of friction coefficient is not included in this study, while preliminary separate numerical analyses suggest that the effect of friction coefficient is minimal for input base motions with peak base accelerations (PBA) less than or equal to 0.12 g and considerable for PBA = 0.24 g. Following the in-flight T-bar test results during centrifuge tests on normally consolidated clays reported by Zhang [45] and Zhang et al. [46], the shear stress limit was set equal to 0.24 σ’_v_ in kPa, where σ’_v_ is the effective overburden pressure of the clay at the depth of interest.

### Clay constitutive model

**Fig 4:**
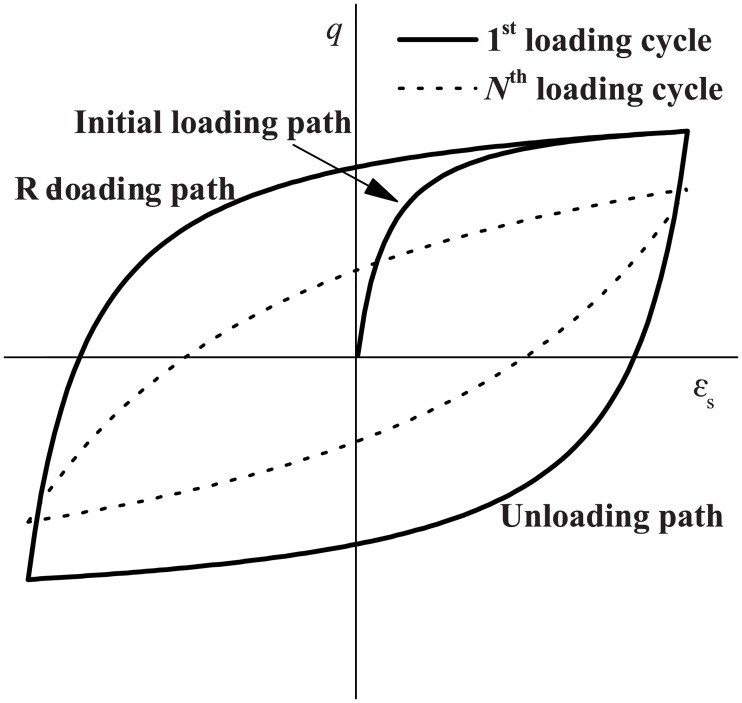
Schematic shear stress-strain relationship for the hyperbolic-hysteretic soil constitutive model (*q* and *ε*_s_ are the current deviatoric stress and generalized shear strain, respectively; *N* is the loading cycle number).

As Fig 4 shows, the normally consolidated clay in this study was modelled by a hyperbolic-hysteretic soil model, which was developed based on a series of test results from cyclic triaxial and resonant column tests on soft clays [44]. The basic shear stress-strain relationship of this model can be expressed by:

$$q=\{\begin{array}{c} q_{f}-\frac{1}{3G_{\text{max}}/q_{f}}\left[\frac{3G_{\text{max}}}{1+3G_{\text{max}}\varepsilon_{s}/q_{f}}\right]\;\text{Initial}\;\text{loading}\;\left(\text{backbone}\right)\;\text{path}\\ -2q_{f}+\frac{2}{3G_{\text{max}}/q_{f}}\left[\frac{3G_{\text{max}}}{1+3G_{\text{max}}\left(\varepsilon_{r1}-\varepsilon_{s}\right)/2q_{f}}\right]+q_{r1}\;\text{Unloading}\;\text{path}\\ 2q_{f}-\frac{2}{3G_{\text{max}}/q_{f}}\left[\frac{3G_{\text{max}}}{1+3G_{\text{max}}\left(\varepsilon_{s}-\varepsilon_{r2}\right)/2q_{f}}\right]-q_{r2}\;\text{Reloading}\;\text{path} \end{array}$$ (1)

where *q* and *ε*_s_ are the current deviatoric stress and generalized shear strain, respectively; *q*_*r*1_ and *q*_*r*2_ are the respective deviatoric stresses at the reversal points; *ε*_r1_ and *ε*_r2_ are the respective generalized shear strains at the reversal points; *G*_max_ is the small-strain shear modulus; *q*_f_ is the deviatoric stress at failure.

The deviatoric stress *q* and generalized shear strain *ε*_s_ can be respectively represented by the following equations:

$$q=\frac{1}{\sqrt{2}}\sqrt{{\left(\sigma_{\text{x}}{}^{\prime }-\sigma_{\text{y}}{}^{\prime }\right)}^{2}+{\left(\sigma_{\text{y}}{}^{\prime }-\sigma_{\text{z}}{}^{\prime }\right)}^{2}+{\left(\sigma_{\text{z}}{}^{\prime }-\sigma_{\text{x}}{}^{\prime }\right)}^{2}+6\left(\tau_{\text{xy}}{}^{2}+\tau_{\text{yz}}{}^{2}+\tau_{\text{zx}}{}^{2}\right)}$$ (2)

$$\varepsilon_{\text{s}}=\frac{2}{3}\sqrt{\frac{1}{2}\left\{{\left(\varepsilon_{\text{x}}-\varepsilon_{\text{y}}\right)}^{2}+{\left(\varepsilon_{\text{y}}-\varepsilon_{\text{z}}\right)}^{2}+{\left(\varepsilon_{\text{z}}-\varepsilon_{\text{x}}\right)}^{2}\right\}+\frac{3}{4}\left(\varepsilon_{\text{xy}}{}^{2}+\varepsilon_{\text{yz}}{}^{2}+\varepsilon_{\text{zx}}{}^{2}\right)}$$ (3)

where *σ*’, *ε* and *τ* respectively denote the effective normal stress, strain and shear stress, with subscript showing the direction.

For normally consolidated clay, following Viggiani and Atkinson [52] and Banerjee [44], the small-strain (or maximum) shear modulus can be expressed by the following formula:

$$G_{\text{max}}=A{\left(p_{0}^{\prime }\right)}^{0.653}$$ (4)

where *p*’_0_ is the initial mean effective normal stress, and the units for both *G*_max_ and *p*’_0_ are kPa; *A* is the calibration constant or stiffness parameter. For kaolin clay, the parameter *A* equals 2060 [44]. This dynamic soil constitutive model was coded into a VUMAT subroutine in ABAQUS for explicit dynamic analysis, which can significantly save the computational time and resource involving seismic soil-structure interaction as compared to the normally used implicit dynamic analysis [51,53]. For example, compared to the implicit dynamic analysis, the computational time involving the present explicit analysis can be significantly reduced by approximately 4 times for the 2D clay-tunnel model considered in this study. More detailed information on this soil model can be found in references [42,44].

## Analysis results

Three influencing factors on the seismic response of clay-tunnel systems were considered in the present study; that is, tunnel wall thickness, ground motion intensity and clay stiffness. The instantaneous profiles of the maximum bending moment and shear force of the tunnel wall correspond to the instant when the tunnel wall attained its respective maximum values. Unless otherwise stated, the parameter *A* is set as 2060.

### Soil stress state around tunnel

**Fig 5:**
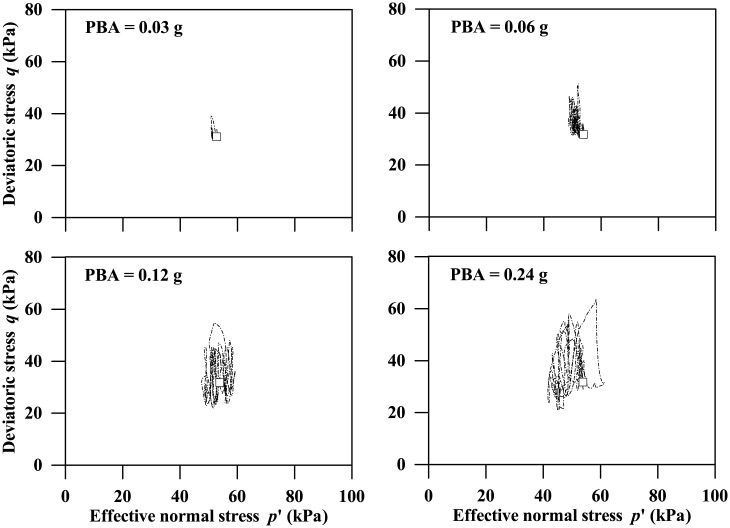
Deviatoric stress versus effective normal stress paths of a clay element neighbouring the left tunnel wall, subjected to the Type-1 base motion (Depth = 12.5 m, Tunnel wall thickness: 0.4 m, open squares designate the initial stress states of the clay element).

**Fig 6:**
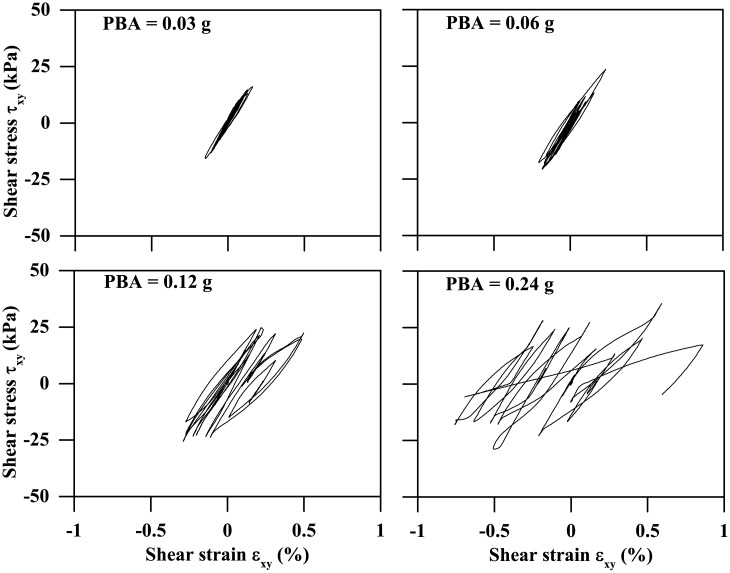
Computed shear stress versus shear strain curves from a clay element neighbouring the left tunnel wall, subjected to the Type-1 base motion (Depth = 12.5 m, Tunnel wall thickness: 0.4 m).

Fig 5 plots the deviatoric stress versus effective normal stress paths for a clay element neighbouring the left tunnel wall, associated with the 0.4-m thickness tunnel subjected to the Type-1 base motions with PBAs ranging from 0.03 g to 0.24 g, where the deviatoric stress was computed using Eq (2). As can be seen, the hysteresis loop and variation in the effective normal stress become more significant with the increasing ground motion intensity. Fig 6 also plots the corresponding shear stress versus shear strain curves, which indicates that the stiffness degradation with increasing shear strain and hysteresis damping can be reasonably captured. This study focuses mainly on the responses of acceleration at different depths and tunnel structure, and in the following subsections the three influencing factors on them are to be investigated.

### Influence of tunnel wall thickness

**Fig 7:**
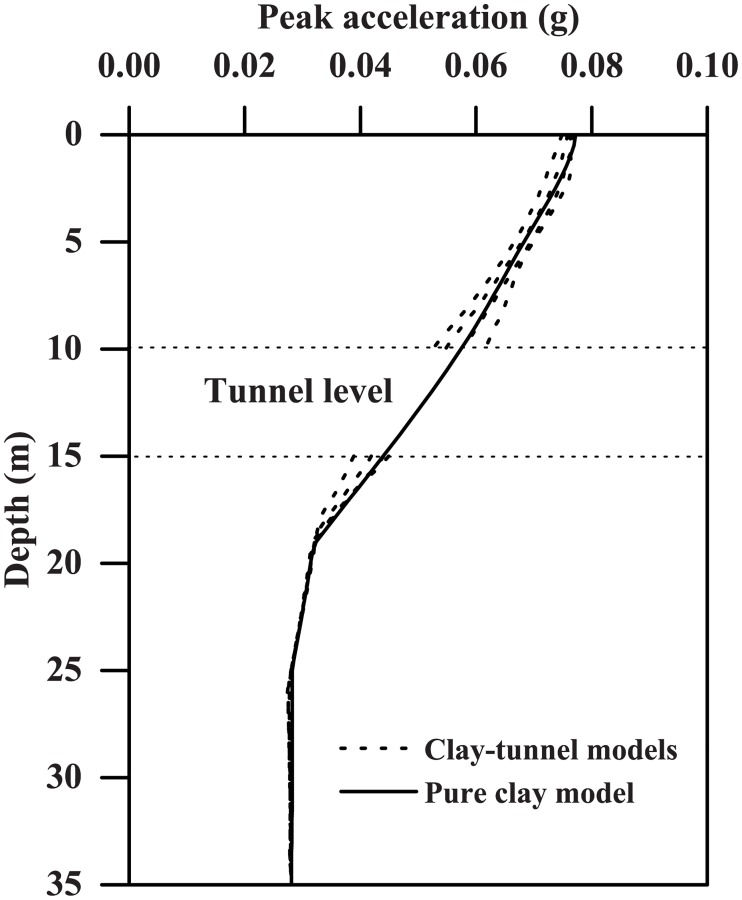
Comparison of the free-field peak ground acceleration profile with those computed from the clay-tunnel models with different wall thicknesses (Type-1 base motion with PBA = 0.03 g).

**Fig 8:**
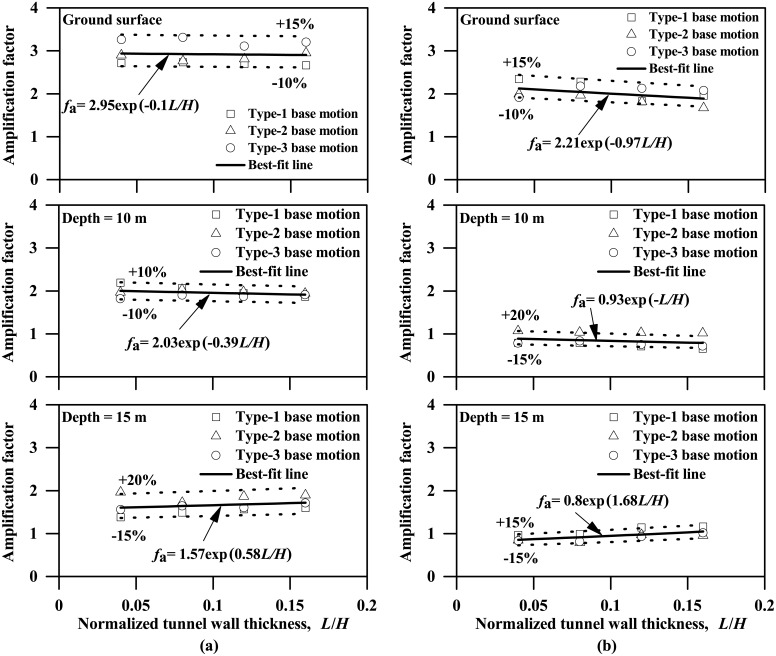
Influence of tunnel wall thickness on the acceleration amplification factors experienced at different depths (*f*_a_: Acceleration amplification factor; *L*, *H* are thickness and height of tunnel wall, respectively). (a) PBA = 0.03 g, (b) PBA = 0.24 g.

Fig 7 shows the peak ground acceleration profiles computed from both the pure clay model and the clay-tunnel models with different wall thicknesses listed in Table 3, subjected to the Type-1 base motion with PBA of 0.06 g. These profiles were obtained by selecting the peak values experienced by the nodes along the symmetrical axis shown in Fig 3. As also reported by many other researchers [39–42], Fig 7 suggests that the clay stratum can significantly amplify the input base motion with a general increasing trend from clay base to the ground surface, regardless of the existence of the tunnel structure or the tunnel wall thickness. In addition, as Fig 7 suggests, the ground acceleration response can be influenced considerably by the dynamic clay-tunnel interaction, especially for the clay nodes located above and underneath (depths equal to 10 m and 15 m, respectively) the tunnel structure, respectively. The influence of dynamic clay-tunnel interaction is also clearly demonstrated in Fig 8, plotting the influence of tunnel thickness on the ground acceleration amplification factors experienced at different depths, where the amplification factor is defined as the ratio of the peak acceleration experienced by a soil node to the peak acceleration of input base motion (i.e. PBA). The acceleration amplification factor against tunnel wall thickness can be represented approximately by the following expressions:

$$\text{For}\;\text{PBA}=\;0.03\text{g}:\;f_{\text{a}}=\{\begin{array}{l} 2.95\;\text{exp}(-0.1L/H)\;\text{Ground}\;\text{surface}\\ 2.03\;\text{exp}(-0.39L/H)\;\text{Above}\;\text{tunnel}\\ 1.57\;\text{exp}(0.58L/H)\;\text{Underneath}\;\text{tunnel} \end{array}$$ (5a)

$$\text{For}\;\text{PBA}=\;0.24\text{g}:\;f_{\text{a}}=\{\begin{array}{l} 2.21\;\text{exp}\left(-0.97L/H\right)\;\text{Ground}\;\text{surface}\\ 0.93\;\text{exp}\left(-L/H\right)\;\text{Above}\;\text{tunnel}\\ 0.8\;\text{exp}\left(1.68L/H\right)\;\text{Underneath}\;\text{tunnel} \end{array}$$ (5b)

where *f*_a_ is the acceleration amplification factor; *L* and *H* are the thickness and height of the tunnel wall, respectively; L/H is the normalized tunnel wall thickness.

The ground acceleration amplification factor at the node underneath tunnel structure against the tunnel thickness shows a general increasing trend, which is reasonable in that a larger portion of the seismic wave tends to be reflected into the soil domain by a stiffer tunnel structure as the wall becomes thicker. However, the opposite slight decreasing trend is found for both the nodes above the tunnel structure and at the ground surface because more ground motion energy is entrapped in the soil domain underneath the tunnel with stiffer walls. The clear discrepancies in the acceleration results shown on Figs 7 and 8 indicate the importance of considering the dynamic soil-tunnel interaction that is also suggested by Ulgen et al. [14] and Tsinidis et al. [15]. Besides, ground amplification effect is generally more significant for input base motion with PBA = 0.03g than that with PBA = 0.24g, while the influence of tunnel wall thickness seems to be more evident associated with PBA = 0.24g. This difference is likely due to that the stiffness and damping ratio associated with the hyperbolic-hysteretic soil model is strain-dependent.

**Fig 9:**
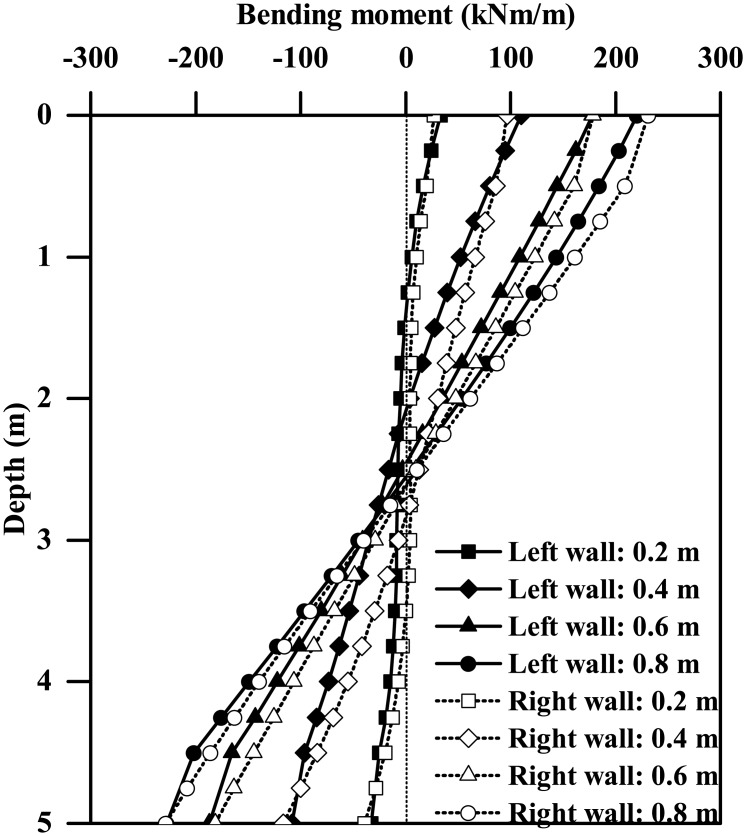
Comparison of the instantaneous maximum bending moment profiles of the tunnel walls with different thicknesses (Type-1 base motion with PBA of 0.03 g).

**Fig 10:**
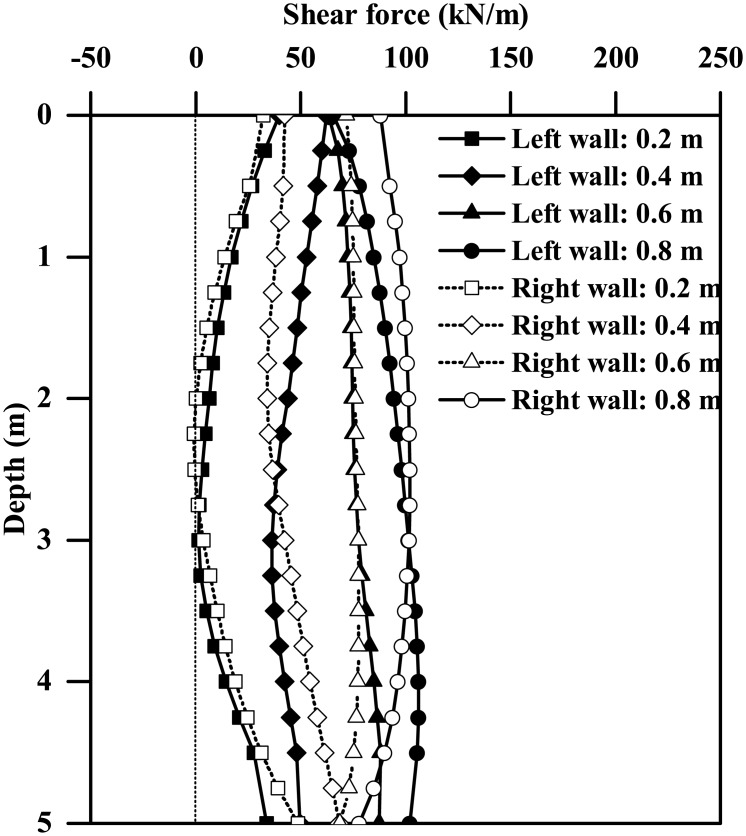
Comparison of the instantaneous maximum shear force profiles of the tunnel walls with different thicknesses (0.03 g PBA Type-1 base motion).

**Fig 11:**
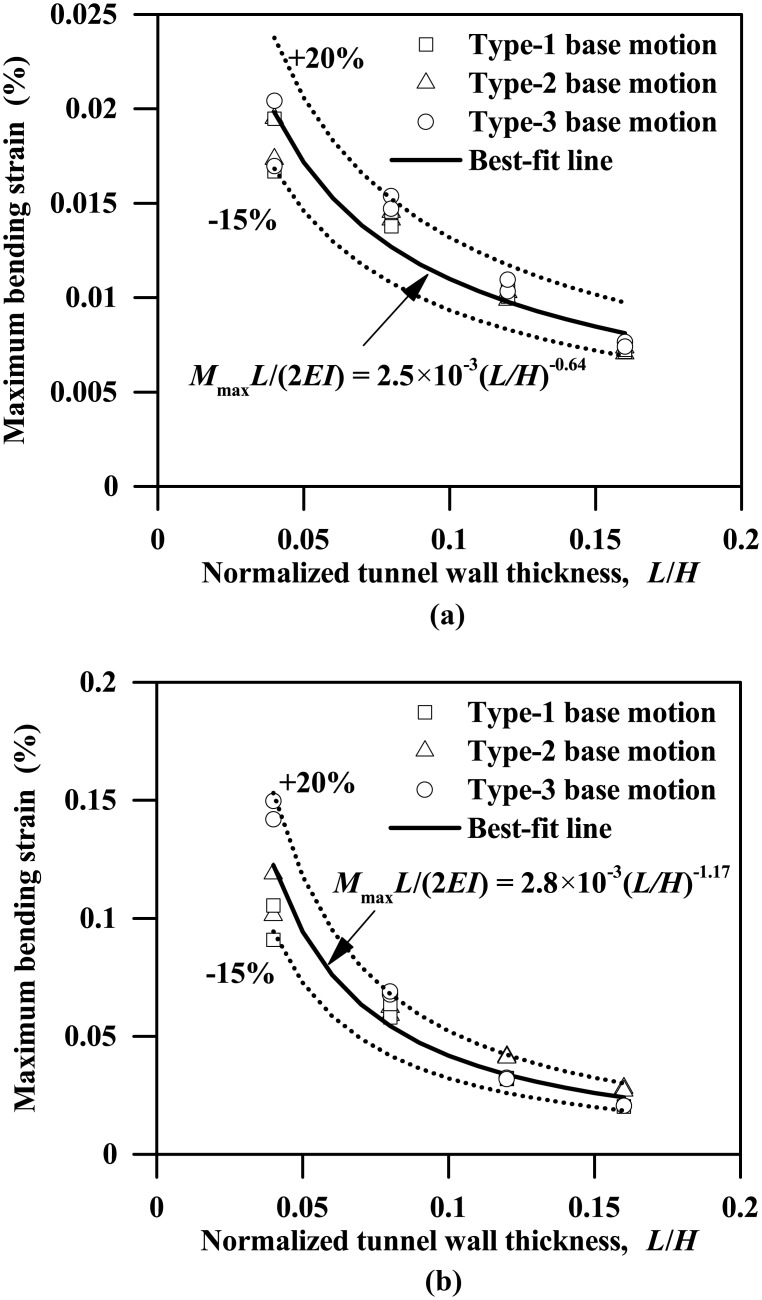
Influence of tunnel wall thickness on the maximum bending strain response of the tunnel wall (*M*_max_: Maximum bending moment experienced by tunnel wall; *EI*: Flexural rigidity of tunnel wall). (a) PBA = 0.03 g, (b) PBA = 0.24 g.

**Fig 12:**
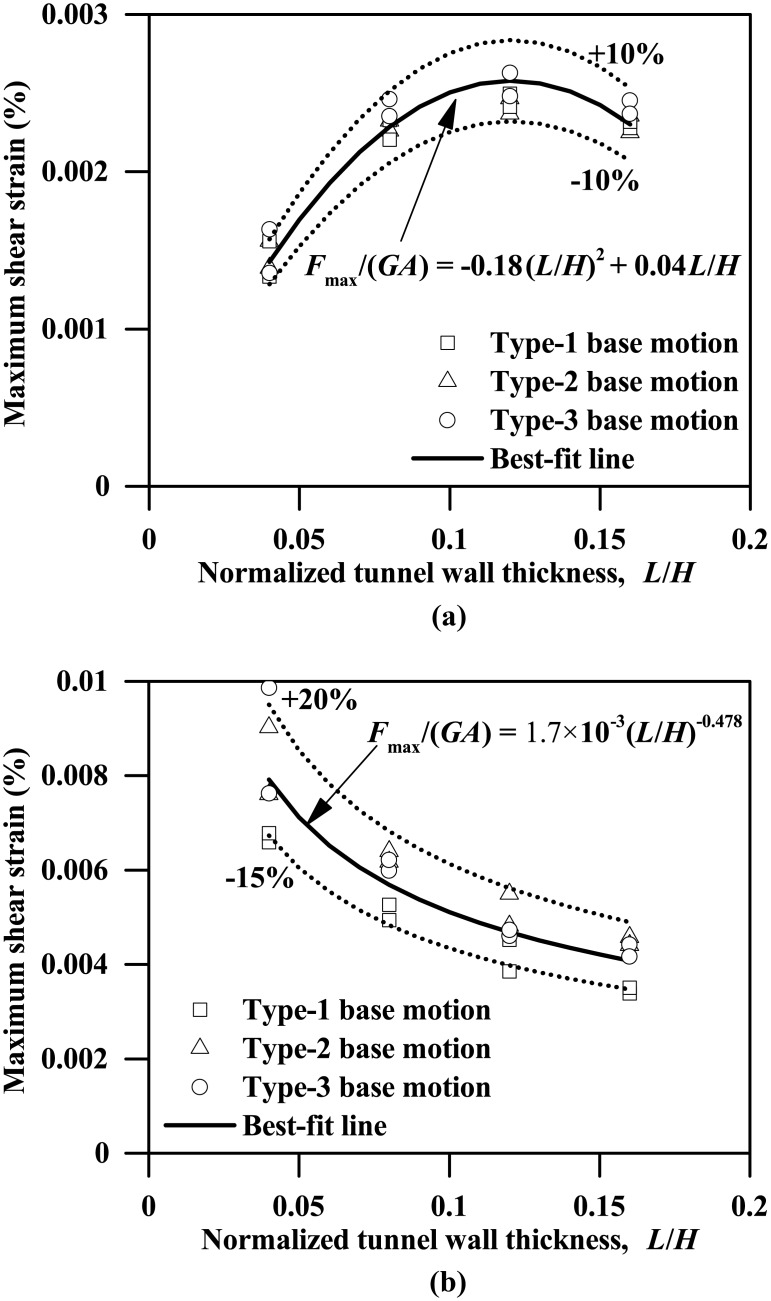
Influence of tunnel wall thickness on the maximum shear strain response of the tunnel wall (*F*_max_: Maximum shear force experienced by tunnel wall, *GA*: Shear rigidity of tunnel wall). (a) PBA = 0.03 g, (b) PBA = 0.24 g.

Fig 9 plots the instantaneous maximum bending moment profiles for both the left and right walls of the tunnels with different wall thicknesses subjected to the 0.03g PBA Type-1 base motion, suggesting a general increasing trend of the maximum bending moment against the wall thickness. The left and right walls generally have different bending moment responses, with the maximum differences of approximately 17% and 2% for tunnel thicknesses equal to 0.2 m and 0.8 m, respectively. Similar trends are also observed for the plots of instantaneous shear force profiles shown in Fig 10. Furthermore, as Figs 11 and 12 show, the maximum bending moment and shear force of the tunnel walls subjected to the three types of base motions can be reasonably well correlated with the tunnel wall thickness by the following equations:

$$\text{For}\;\text{PBA}=\;0.03\text{g}:\;M_{\text{max}}L/\left(2EI\right)=2.5\times 10^{-3}{\left(L/H\right)}^{-0.64}$$ (6a)

$$\text{For}\;\text{PBA}=\;0.24\text{g}:\;M_{\text{max}}L/\left(2EI\right)=2.8\times 10^{-3}{\left(L/H\right)}^{-1.17}$$ (6b)

$$\text{For}\;\text{PBA}=\;0.03\text{g}:\;F_{\text{max}}/\left(GA\right)=-0.18{\left(L/H\right)}^{2}+0.04L/H$$ (7a)

$$\text{For}\;\text{PBA}=\;0.24\text{g}:\;F_{\text{max}}/\left(GA\right)=1.7\times 10^{-3}{\left(L/H\right)}^{-0.478}$$ (7b)

where *M*_max_ and *F*_max_ are the respective maximum bending moment and shear force of the tunnel wall; *EI* and *GA* are the flexural rigidity and shear rigidity of the tunnel wall, respectively; *M*_max_*L*/(2*EI*) and *F*_max_/(*GA*) are the maximum bending strain and maximum shear strain experienced by the tunnel wall, in percentage terms.

**Fig 13:**
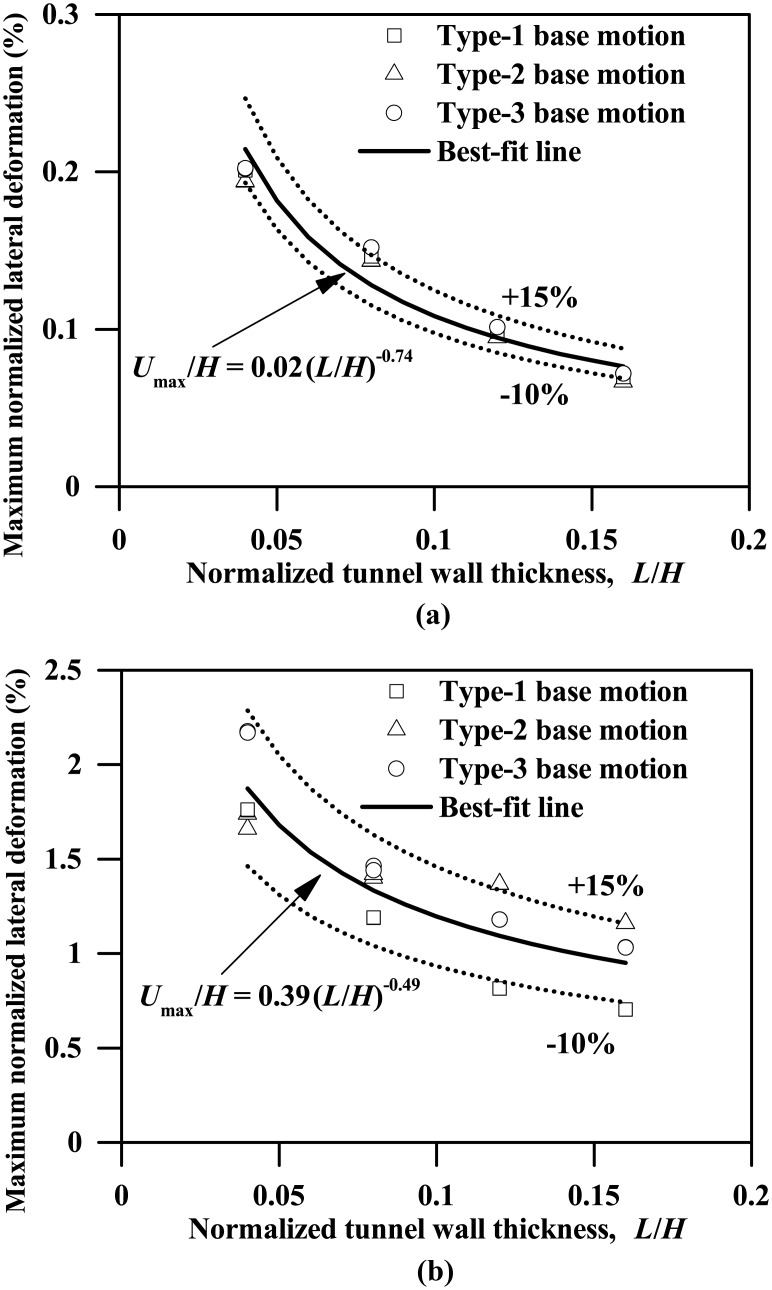
Influence of tunnel wall thickness on the maximum normalized lateral deformation response of the tunnel wall (*U*_max_: Maximum lateral deformation of tunnel wall). (a) PBA = 0.03 g, (b) PBA = 0.24 g.

The instantaneous maximum lateral deformation profiles of the tunnel walls, which generally follow a relatively simple trend and can be approximately represented by a liner line, are not presented herein. Instead, the results of the maximum lateral deformation of the tunnel wall, which is defined as the maximum relative lateral displacement between the two ends of the tunnel wall throughout the respective seismic shaking event, are presented in this study. Fig 13 shows the plots of the maximum normalized lateral deformation of the lateral wall against the normalized tunnel wall thickness for clay-tunnel systems subjected to the three types of base motions with PBAs of 0.03g and 0.24g, which suggests that the relationship between the maximum lateral deformation of the tunnel wall and the tunnel wall thickness can be approximately represented by the following equations:

$$\text{For}\;\text{PBA}=\;0.03\text{g}:\;U_{\text{max}}/H=0.02{\left(L/H\right)}^{-0.74}$$ (8a)

$$\text{For}\;\text{PBA}=\;0.24\text{g}:\;U_{\text{max}}/H=0.39{\left(L/H\right)}^{-0.49}$$ (8b)

where *U*_max_ is the maximum lateral deformation of the tunnel wall; *U*_max_ /H is maximum normalized lateral deformation of the tunnel wall, in terms of percentage.

Furthermore, Figs 11–13 show that the influences of tunnel wall thickness on the seismic response of tunnel structure are generally different for base motions with varying PBAs, indicating that the seismic clay-tunnel interaction depends on the ground motion intensity. In the next section, more computed results pertaining to the effect of ground motion intensity on the clay-tunnel systems are to be reported.

### Influence of ground motion intensity

**Fig 14:**
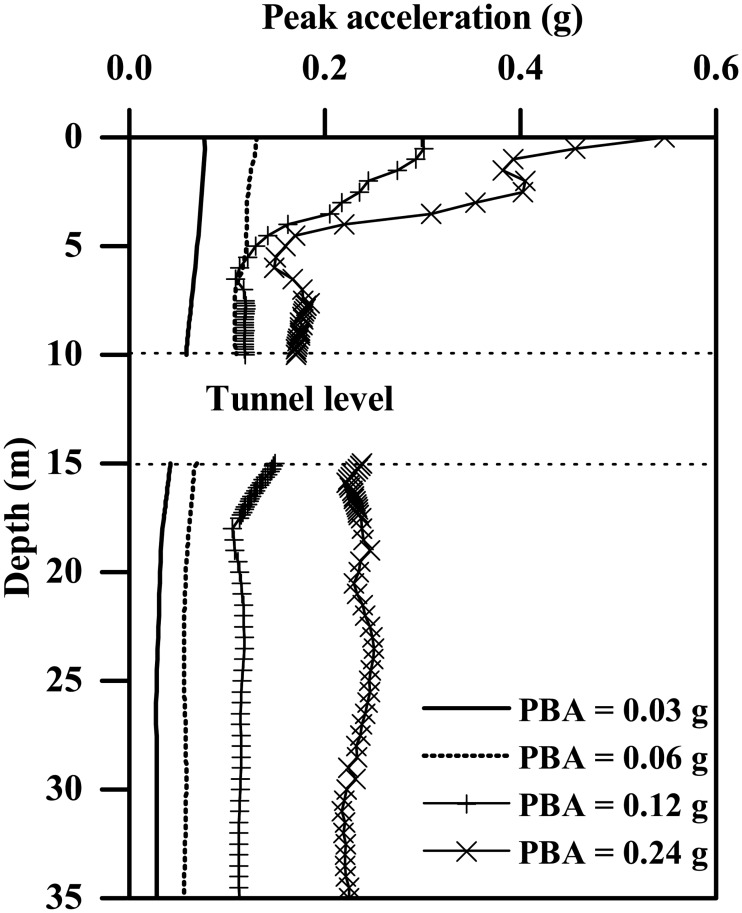
Comparison of the peak ground acceleration profiles for the clay-tunnel model subjected to different-PBA base motions (*L* = 0.4 m, Type-1 base motions).

**Fig 15:**
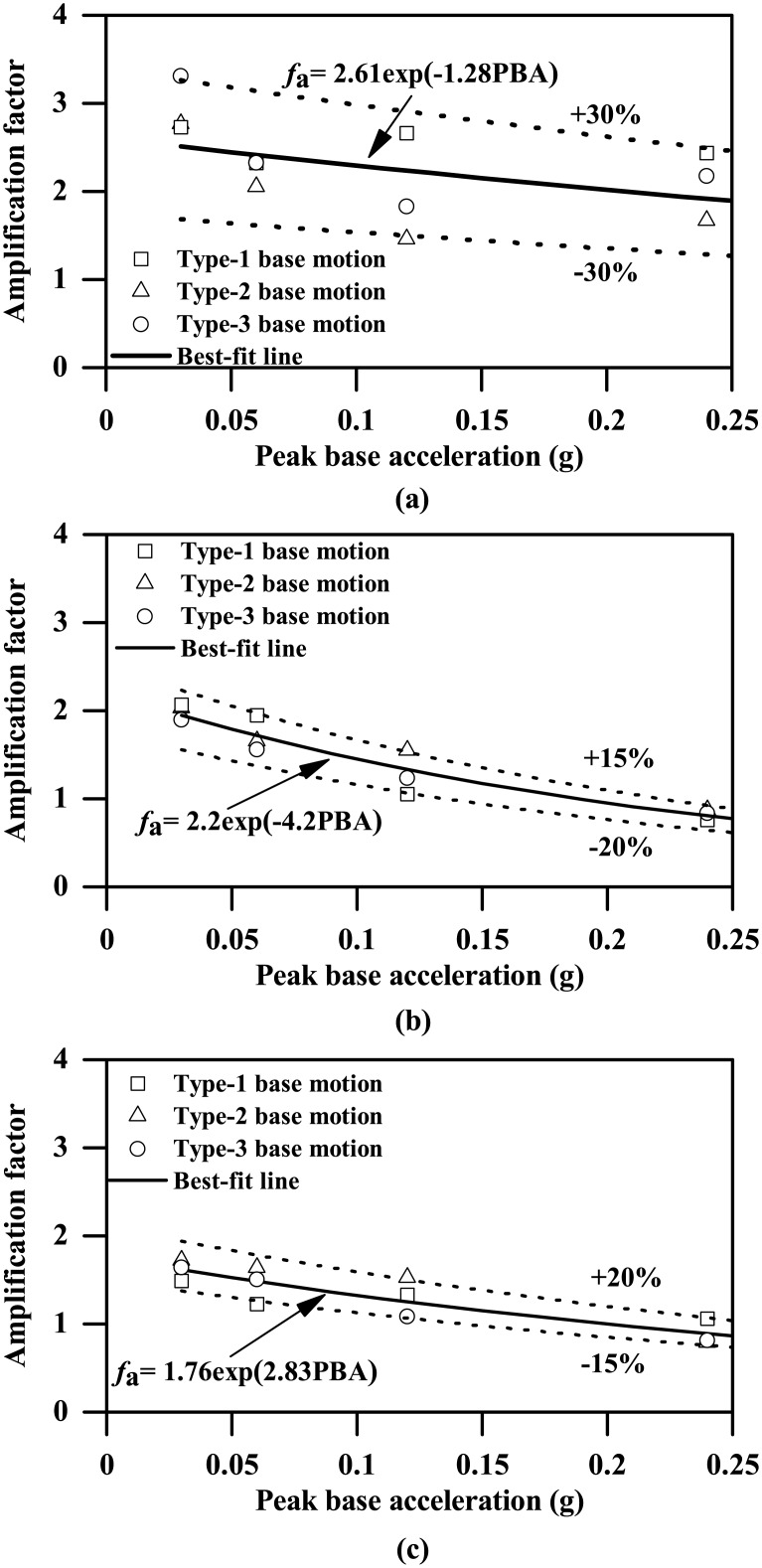
Influence of PBA on acceleration amplification factors experienced at different depths (*L* = 0.4 m). (a) ground surface, (b) depth = 10 m, (c) depth = 15 m.

Four different-intensity ground motions are used to study the effect of ground motion intensity, the PBAs ranging from 0.03g to 0.24g, as shown in both Fig 1 and Table 3. As Fig 14 shows, ground acceleration response can also be significantly influenced by the PBA, with a clearly increasing trend. In particular, accelerations experienced at the location underneath the tunnel structure seem to be more sensitive to the variation in PBA, which are generally smaller than those experienced above the tunnel for PBAs less than or equal to 0.06g and then become comparatively larger for PBAs of 0.12g and 0.24g. Fig 15 shows the plots of the acceleration amplification factor against PBA at the different depths, which suggest a general decreasing trend. In addition, the acceleration amplification factors against PBA can be reasonably well represented by the following expression:

$$f_{\text{a}}=\{\begin{array}{l} 2.61\;\text{exp}\left(-1.28\text{PBA}/g\right)\;\text{Ground}\;\text{surface}\\ 2.2\;\text{exp}\left(-4.2\text{PBA}/g\right)\;\text{Above}\;\text{tunnel}\\ 1.76\;\text{exp}\left(2.83\text{PBA}/g\right)\;\text{Underneath}\;\text{tunnel} \end{array}$$ (9)

where *g* is the gravitational acceleration, and PBA/g is the normalized PBA.

**Fig 16:**
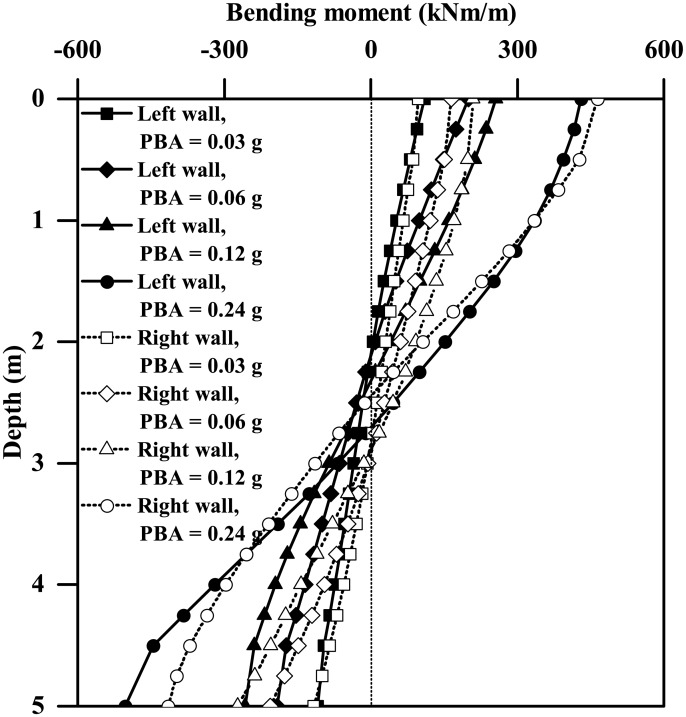
Comparison of the instantaneous maximum bending moment profiles of the tunnel walls subjected to different-PBA base motions (*L* = 0.4 m, Type-1 base motions).

**Fig 17:**
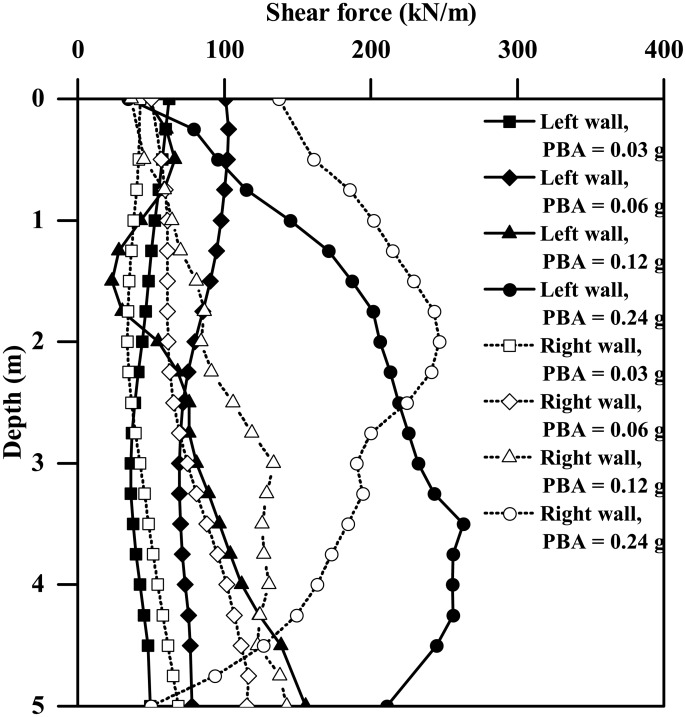
Comparison of the instantaneous maximum shear force profiles of the tunnel walls subjected to different-PBA base motions (*L* = 0.4 m).

**Fig 18:**
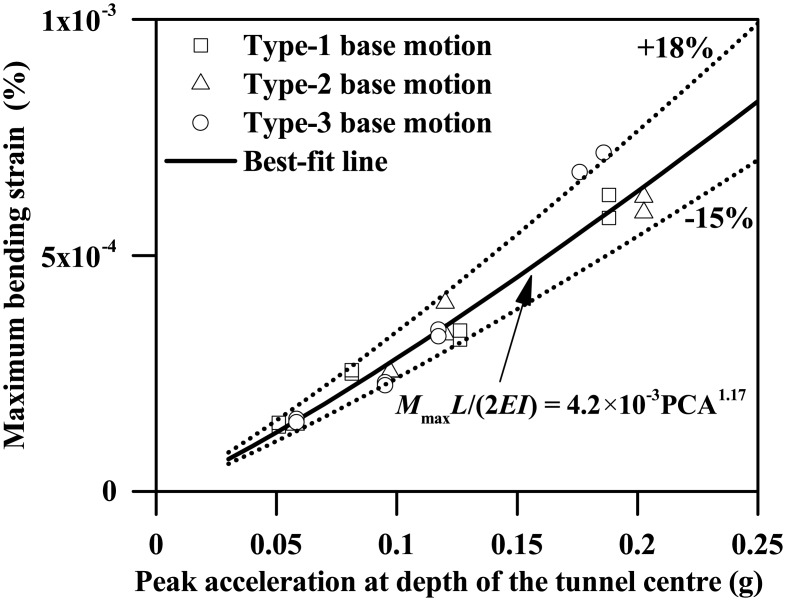
Plot of the maximum bending strain response of the tunnel wall against the free-field peak acceleration at depth of the tunnel centre (PCA) (*L* = 0.4 m).

**Fig 19:**
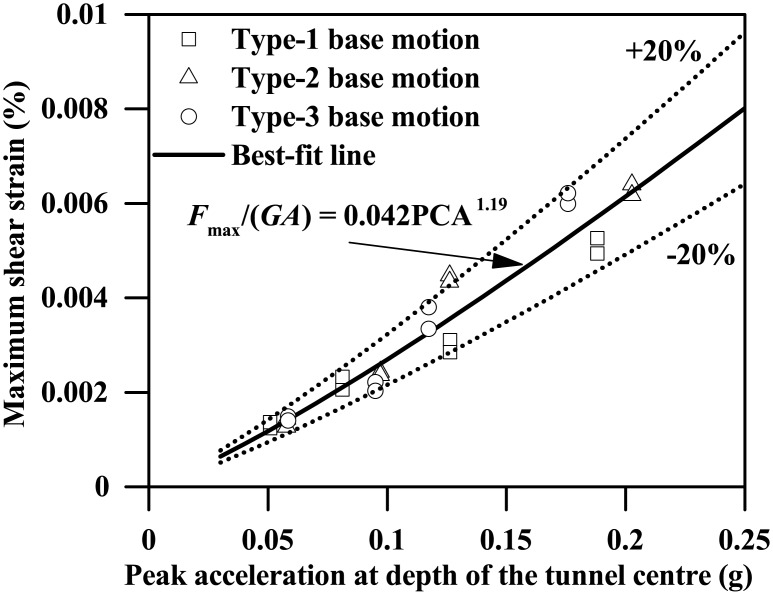
Plot of the maximum shear strain response of the tunnel wall against the free-field peak acceleration at depth of the tunnel centre (*L* = 0.4 m).

As Figs 16 and 17 show, both the maximum bending moment and the shear force of the tunnel wall increase monotonically with the increasing PBA, the lateral and right walls generally having different responses. Like Fig 9, the maximum values of the bending moment shown in Fig 16 are generally experienced at the location of tunnel wall top or base. Similar findings were also observed for the shear force response of the tunnel wall for PBAs less than or equal to 0.12g, as Fig 17 shows. However, for a PBA of 0.24g, the maximum shear force of the tunnel wall can be experienced at the middle depth or lower half depth along the tunnel wall, which probably indicates the occurrence of separation between the clay and the tunnel wall at the clay-tunnel interface and a crossover point of the distribution of dynamic stress arising from the surrounding clays. The earthquake-induced kinematic force upon the tunnel structure is highly dependent on the motion intensities of the surrounding soils. In this study, the free-field peak acceleration at depth of the tunnel centre (depth = 12.5 m), termed PCA, is used to represent the average motion intensity of the soils at the tunnel level. As Figs 18 and 19 show, the maximum bending moment and the shear force of the tunnel wall can be well correlated with the PCA by the following two equations:

$$M_{\text{max}}L/\left(2EI\right)=4.2\times 10^{-3}{\left(\text{PCA}/g\right)}^{1.17}$$ (10)

$$F_{\text{max}}/\left(GA\right)=0.042{\left(\text{PCA}/g\right)}^{1.19}$$ (11)

where PCA/*g* is the normalized PCA.

**Fig 20:**
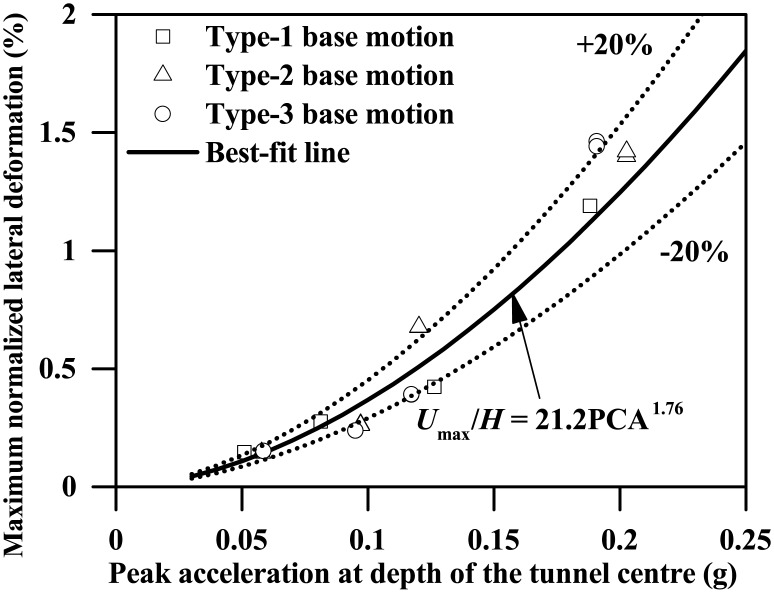
Plot of the maximum normalized lateral deformation of the tunnel wall against the free-field peak acceleration at depth of the tunnel centre (*L* = 0.4 m).

Furthermore, Fig 20 also plots the maximum normalized lateral deformation of the tunnel wall against the PCA, which suggests a good relationship between them, as shown below.

$$U_{\text{max}}/H=21.2{\left(\text{PCA}/g\right)}^{1.76}$$ (12)

As Figs 14 and 16–20 show, the ground motion intensity generally has a clear increasing trend of influence on the seismic response of the tunnel structure and the ground acceleration response. However, the ground acceleration amplification factors (Fig 15) experienced at different depths tend to gradually decrease with the increasing ground motion intensity, which is likely due to the nonlinear stress-strain relationship, stiffness reduction and damping augmentation with the increasing strain level associated with the adopted hyperbolic-hysteretic clay model.

### Influence of clay stiffness

The influence of the clay stiffness was considered by varying the parameter *A* in Eq (4), the values of which are listed in Table 3. Due to space constraints, the profiles of the peak ground acceleration, the maximum bending moment and the shear force of the tunnel wall associated with different clay shear moduli are not presented herein.

**Fig 21:**
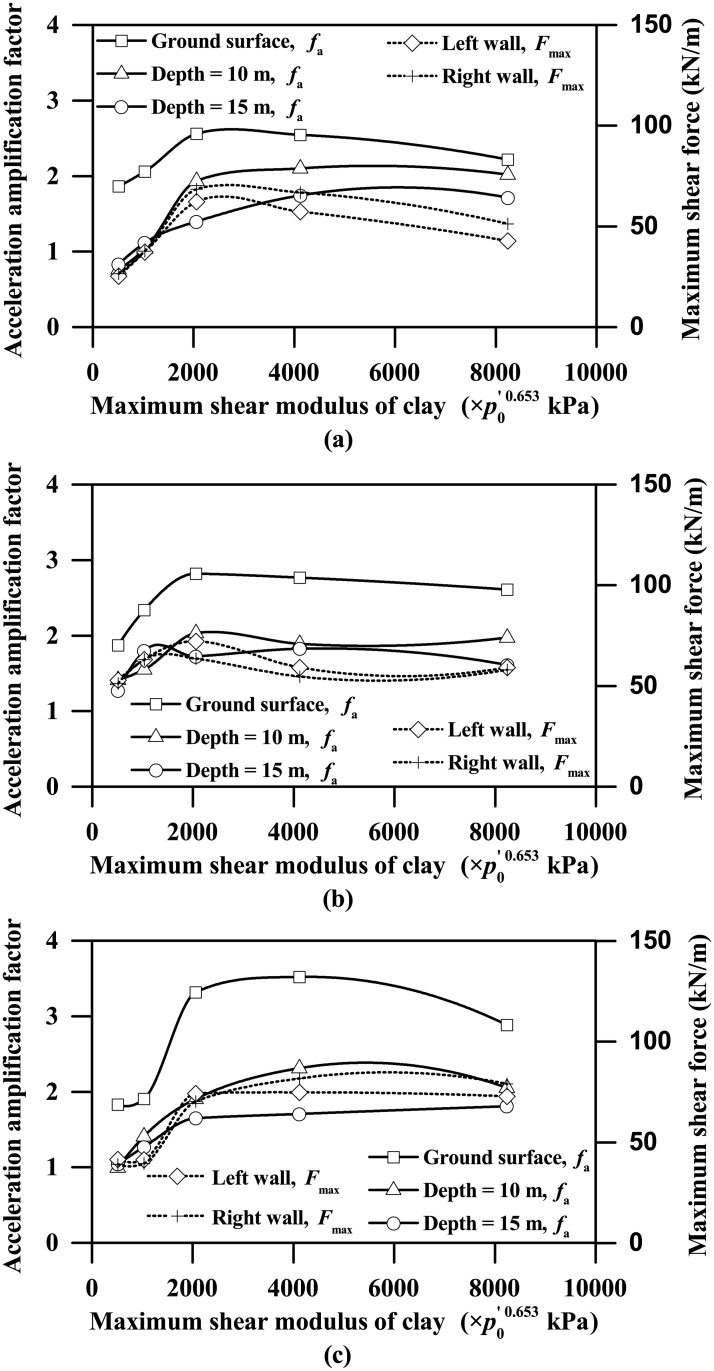
Influence of clay stiffness on acceleration amplification factor and maximum shear force responses of the tunnel wall (*p*_0_’ = initial mean effective normal stress of the clay; *L* = 0.4 m; PBA = 0.03 g). (a) Type-1 base motion, (2) Type-2 base motion, (3) Type-3 base motion.

**Fig 22:**
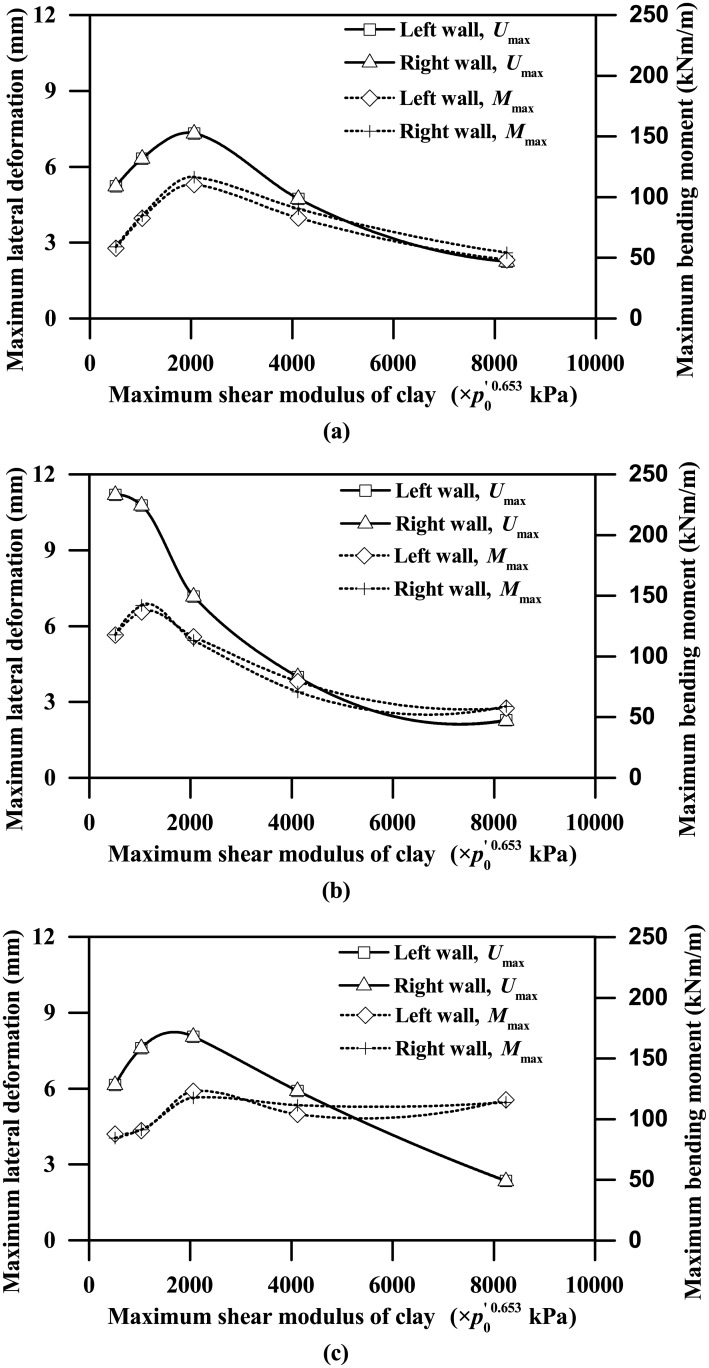
Influence of clay stiffness on maximum lateral deformation and bending moment responses of the tunnel wall (*L* = 0.4 m; PBA = 0.03 g). (a) Type-1 base motion, (2) Type-2 base motion, (3) Type-3 base motion.

Fig 21 shows the trends of the acceleration amplification factor and maximum shear force versus the maximum shear modulus of clay. Both the acceleration amplification factor and the maximum shear force tend to increase steadily with the increasing clay stiffness up to a critical value, beyond which they tend to have the opposite trends or remain relatively unchanged with the increasing clay stiffness. Similar findings are also observed in Fig 22, showing the trends of the maximum lateral deformation and bending moment against the clay stiffness. For the plots shown in Figs 21(a) and 22(a) involving the Type-1 base motion, the critical value of the maximum shear modulus of clay can be expressed approximately by the following equation.

$$G_{\text{max}}=2060{\left(p_{0}^{\prime }\right)}^{0.653}$$ (13)

According to Banerjee [44] and Banerjee et al. [42], Eq (13) can well represent the maximum shear modulus of normally consolidated Malaysian kaolin clay.

**Fig 23:**
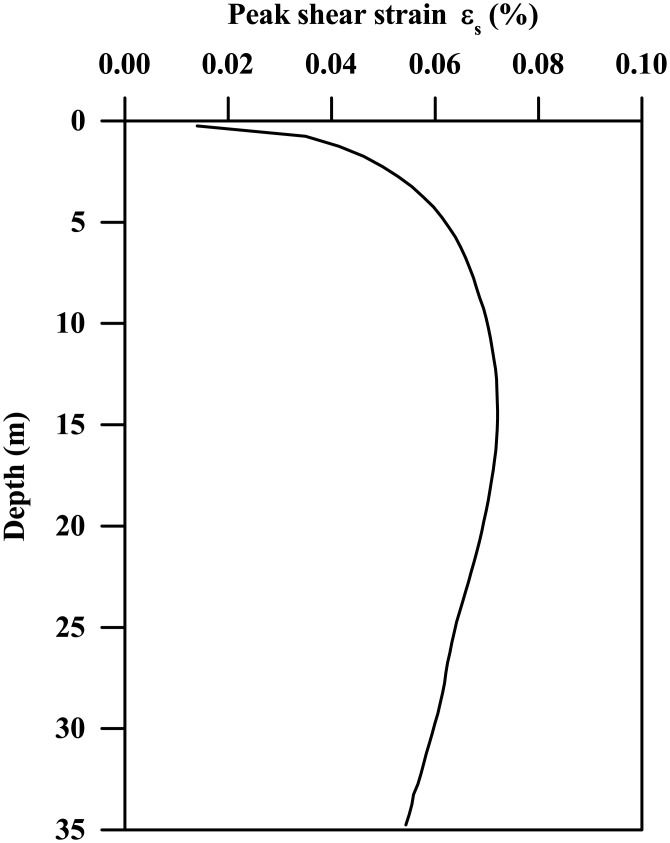
Peak shear strain profile along the clay depth for pure clay stratum subjected to the Type-1 base motion with PBA of 0.03 g (stiffness parameter = 2060).

To better understand the trends demonstrated in Figs 21 and 22, it is helpful to compare the fundamental period of the clay stratum with the dominant period of the input base motion. Fig 23 plots the typical peak shear strain profile along the clay depth for pure clay bed subjected to the Type-1 base motion with PBA of 0.03 g. As can be seen, the peak shear strains are generally very small, with the maximum value less than 0.08%. For such small shear strain levels, the stiffness degradation effect is insignificant and can be neglected, which is also demonstrated in Fig 6(a). Hence, the fundamental period calculated based on the maximum soil shear modulus is approximately the same as that of clay stratum subjected to small base motions with PBA of 0.03g.

The depth-averaged maximum soil shear modulus *G*_soil_ may be represented by the following equation:

$$G_{\text{soil}}=\frac{\int_{0}^{H_{\text{soil}}}G_{\text{max}}dz}{H_{\text{soil}}}$$ (14)

where *H*_soil_ is the thickness of the clay from the ground surface to the clay base, with the unit of m; *G*_max_ is the depth-dependent maximum and can be calculated based on Eq (4) and the stiffness parameters listed in Table 3.

Substituting the critical clay stiffness represented by Eq (13) into Eq (14) leads to:

$$G_{\text{soil}}\approx 3235\times {\left(H_{\text{soil}}\right)}^{0.653}$$ (15)

According to Kramer [54], the fundamental period of a soil stratum can be estimated using the following equation:

$$T_{\text{soil}}=\;4H_{\text{soil}}/V_{\text{s}}$$ (16)

where *T*_soil_ is the fundamental period of a soil stratum, with the units of s; *V*_s_ is the equivalent shear wave velocity in a soil stratum, with the units of m/s, which can be obtained following the equation:

$$V_{\text{s}}=\;{\left(G_{\text{soil}}/\rho_{\text{soil}}\right)}^{0.5}$$ (17)

where *ρ*_soil_ is the density of soil, with the units of kg/m^3^.

**Fig 24:**
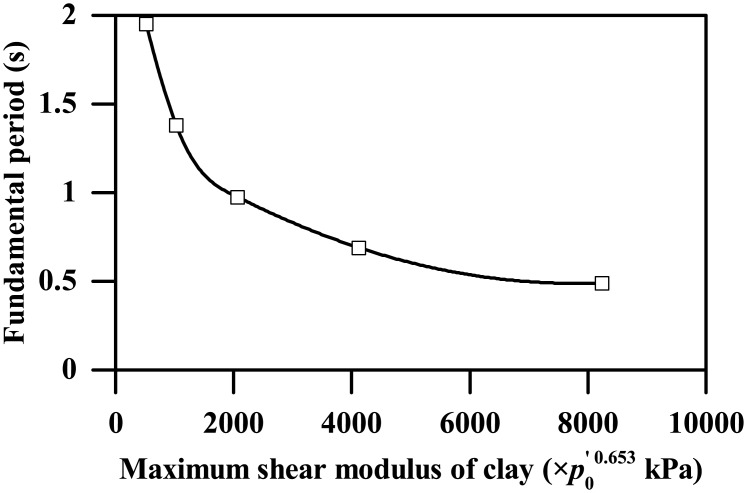
Plot of the fundamental period of the clay stratum versus its maximum shear modulus.

By substituting the soil thickness and stiffness parameters into Eq (16), the fundamental periods of the clay deposits corresponding to different maximum shear moduli can be obtained, as shown in Fig 24.

Fig 24 shows that, for maximum shear modulus of clay less than the critical shear modulus corresponding to the Type-1 base motion, the fundamental period of the soil stratum decreases from approximately 2 s to 1 s, which gradually becomes closer to the dominant period (approximately 0.9 s, as Fig 1 shows) of the Type -1 base motion. Beyond the critical shear modulus, the fundamental period of the soil stratum continues to decrease gradually until down to approximately 0.5 s corresponding to the stiffness parameter *A* equal to 8240, which has a trend of augmenting the difference with the dominant period of the input base motion. In general, smaller differences between the fundamental period of the soil stratum and the dominant period of the input base motion lead to more intense seismic response, and vice versa. Hence, the trends shown in Figs 21 and 22 of both the ground acceleration and the tunnel structure responses against the clay stiffness are highly frequency-dependent and can be reasonably well explained by comparing the fundamental period of the clay deposit with the dominant period of the input base motion.

### Comparison with the existing analytical approaches

As mentioned in Section Introduction, the experimental and numerical data of the seismic behavior of rectangular tunnel installed in soft clay deposits is very limited. In this study, the computed racking deformation results of the tunnel structure are compared with the estimations of analytical approaches developed by Wang [5], Penzien [6] and Anderson et al. [11].

For relatively stiff tunnels where the rotation of the tunnel structure can be neglected, the maximum lateral deformation of the tunnel wall is also termed racking deformation or distortion [5–6]. However, as suggested by Debiasi et al. [33] and Ulgen et al. [26], for relatively flexible tunnel structures, the lateral deformation component due to rocking rotation should be subtracted from the maximum lateral deformation to obtain the racking deformation of tunnel structure. In this study, the effect of tunnel rotation on the maximum lateral deformation of the tunnel wall was found to be insignificant and hence neglected. The racking ratio of a tunnel can be represented by the following equation:

$$R=\Delta_{\text{str}}/\Delta_{\text{ff}}$$ (18)

where *R* is the racking ratio of a tunnel, Δ_str_ is the racking distortion of a tunnel, and Δ_str_ is the free-field ground distortion between the depths, respectively equal to tunnel top and bottom depths.

The racking ratio is usually correlated with the flexibility ratio *F*, which is expressed by the following equation:

$$F=G_{\text{s}}\times W/(S\times H)$$ (19)

where *W* is the tunnel width, H is the tunnel height, *S* is the force required to cause a unit racking deflection of the tunnel, computed through a simple static elastic frame analysis, and G_s_ is the soil shear modulus.

For the hyperbolic-hysteretic soil model used in this study, a degraded soil shear modulus should be used for *G*_s_, which can be estimated using the following equation [44]:

$$G_{s}=\frac{G_{\text{max}}}{1+\frac{3G_{\text{max}}}{q_{\text{f}}}\varepsilon_{\text{s}}}$$ (20)

**Fig 25:**
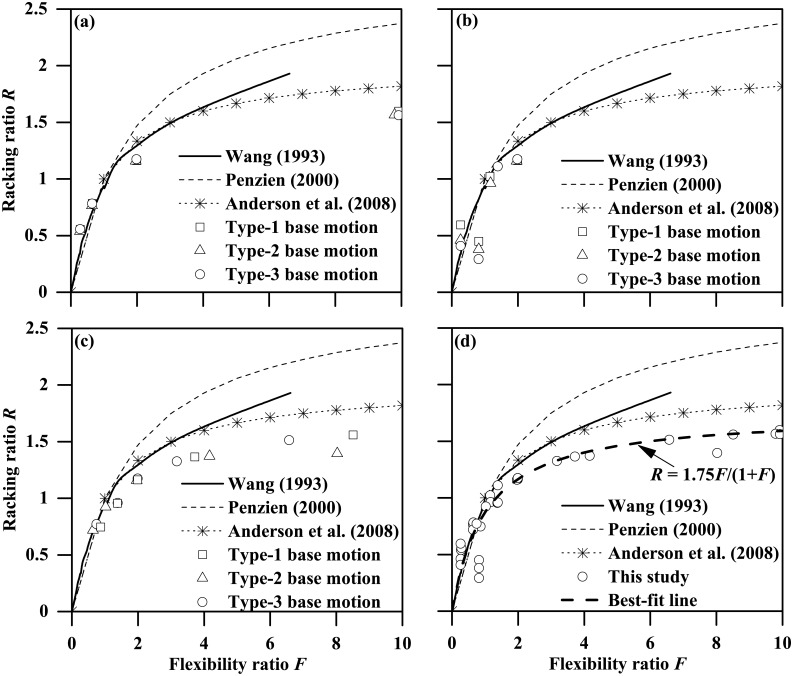
Racking ratio against flexibility ratio plots obtained using analytical approaches and from this study. (a) *L* = 0.2 m to 0.8 m (PBA = 0.03 g), (b) PBAs = 0.03 g to 0.24 g (*L* = 0.4 m), (c) clay stiffness parameters = 515 to 8240 (PBA = 0.03 g; *L* = 0.4 m), (d) combined data from (a)-(c).

Fig 25 presents plots of the racking ratio against the flexibility ratio obtained from the present FE analyses and using analytical approaches developed by Wang [5], Penzien [6] and Anderson et al. [11]. As can be seen, for flexibility ratios less than about 1.5, notwithstanding the local discrepancies, the overall trend of the racking ratio against the flexibility ratio obtained from this study compares favorably well with these suggested by the analytical solutions. On the other hand, for flexibility ratios larger than 2, all the three analytical approaches tend to considerably overestimate the racking ratio of tunnel structure embedded in soft clay deposit, with the closest prediction given by Anderson et al. [11]. Furthermore, as Fig 25(d) shows, the computed racking ratio versus the flexibility ratio in this study can be well represented by the following expression:

R=1.75F/(1+F) (21)

Eq (21) is useful in estimating the racking distortion of a rectangular tunnel embedded in soft deposits subjected to seismic ground motions. It can be estimated that the predictions of tunnel racking ratio using Eq (21) are about 12.5% less than that suggested by Anderson et al. [11].

## Conclusions

A series of 2D FE parametric studies incorporated with a hyperbolic-hysteretic soil constitutive model were performed to investigate the seismic response of rectangular tunnels installed in clay strata subjected to three types of far-field ground motions, in which three crucial factors, namely tunnel wall thickness, peak base acceleration and clay stiffness, were considered. All the three factors have significant influence on both the responses of ground acceleration and tunnel structure. Some specific conclusions are summarized below.

1. Due to the effect of clay-tunnel interaction, the ground acceleration response with the presence of tunnel structure is significantly different than the free-field ground acceleration response, especially for the locations at the depths near the tunnel level.
2. The soft clay deposits can significantly amplify the earthquake-induced ground motions, regardless of the values of tunnel wall thickness or PBA, with the maximum acceleration amplification factor found to be larger than 3 (as shown in Figs 8a and 15a).
3. The influence of clay stiffness on the seismic response of the clay-tunnel system is highly frequency-dependent. In the future study, random FE analyses (e.g. Zhang et al. [55], Liu and Zhang [56]) may be employed to better investigate the influences of soil properties on the seismic response of the clay-tunnel system, which may facilitate the reliability-based design for tunnels installed in clay deposits.
4. Some useful correlations are formulated using dimensionless terms to relate the maximum seismic responses of clay-tunnel systems to each of the influencing factors considered, which may provide a useful reference for the practical seismic design of tunnels installed in soft clay deposits.
5. The existing analytical approaches developed by Wang [5], Penzien [6] and Anderson et al. [11] can favourably predict the racking deformation response of rectangular tunnel installed in soft clay deposits for flexibility ratios less than 1.5, beyond which these approaches tend to significantly overestimate the racking deformation response. In view of this, a new semi-empirical equation to correlate the racking ratio with flexibility ratio for rectangular tunnels embedded in soft clay deposits subjected to seismic shakings is derived, with the aid of regression analysis using the present FE analysis results.

The present study focuses on the seismic performance of clay-tunnel systems subjected to far-field ground motions. Hence, the findings and correlations presented in this study may be valid only for rectangular tunnels installed in soft clay deposits subjected to the earthquakes with the frequency contents comparable to that adopted in this study, and care should be exercised when applying the findings drawn in this study to other types of earthquakes. Future studies can be performed to further examine the seismic behaviour of the clay-tunnel systems subjected to different earthquakes with distinct dominant periods.

## Supporting information

S1 File Original data and plot for Fig 1 in the article. (OPJ)

S2 File Original data and plot for Fig 2 in the article. (OPJ)

S3 File Original data and plot for Fig 4 in the article. (OPJ)

S4 File Original data and plot for Fig 5 in the article. (OPJ)

S5 File Original data and plot for Fig 6 in the article. (OPJ)

S6 File Original data and plot for Fig 7 in the article. (OPJ)

S7 File Original data and plot for Fig 8a in the article. (OPJ)

S8 File Original data and plot for Fig 8b in the article. (OPJ)

S9 File Original data and plot for Fig 9 in the article. (OPJ)

S10 File Original data and plot for Fig 10 in the article. (OPJ)

S11 File Original data and plot for Fig 11a in the article. (OPJ)

S12 File Original data and plot for Fig 11b in the article. (OPJ)

S13 File Original data and plot for Fig 12a in the article. (OPJ)

S14 File Original data and plot for Fig 12b in the article. (OPJ)

S15 File Original data and plot for Fig 13a in the article. (OPJ)

S16 File Original data and plot for Fig 13b in the article. (OPJ)

S17 File Original data and plot for Fig 14 in the article. (OPJ)

S18 File Original data and plot for Fig 15 in the article. (OPJ)

S19 File Original data and plot for Fig 16 in the article. (OPJ)

S20 File Original data and plot for Fig 17 in the article. (OPJ)

S21 File Original data and plot for Fig 18 in the article. (OPJ)

S22 File Original data and plot for Fig 19 in the article. (OPJ)

S23 File Original data and plot for Fig 20 in the article. (OPJ)

S24 File Original data and plot for Fig 21 in the article. (OPJ)

S25 File Original data and plot for Fig 22 in the article. (OPJ)

S26 File Original data and plot for Fig 23 in the article. (OPJ)

S27 File Original data and plot for Fig 24 in the article. (OPJ)

S28 File Original data and plot for Fig 25 in the article. (OPJ)

## References

[1] Iida H, Hiroto T, Yoshida N, Iwafuji M. Damage to Daikai subway station. Soils and Foundations. 1996; 36(Special): 283–300.

[2] Wang WL, Wang TT, Su JJ, Lin CH, Seng CR, Huang TH. Assessment of damage in mountain tunnels due to the Taiwan Chi-Chi earthquake. Tunnelling and Underground Space Technology. 2001; 16(3): 133–150.

[3] Hashash YMA, Hook JJ, Schmidt B, Yao JIC. Seismic design and analysis of underground structures. Tunnelling and Underground Space Technology. 2001; 16(4): 247–293.

[4] Kontoe S, Zdravkovic L, Potts DM, Menkiti CO. Case study on seismic tunnel response. Canadian Geotechnical Journal. 2008; 45(12): 1743–1764.

[5] Wang JN. Seismic design of tunnels: a simple state of the art design approach. Parsons Brinckerhoff Inc., New York 1993.

[6] Penzien J. Seismically induced racking of tunnel linings. Earthquake Engineering and Structural Dynamics. 2000; 29(5): 683–691.

[7] NSPRC (National Standard of the People’s Republic of China). JTGD70-2004: Code for design of road tunnel. Ministry of Transport of the People’s Republic of China, Beijing. 2004.

[8] ISO (International Organization for Standardization). ISO 23469: Bases for design of structures—seismic actions for designing geotechnical works. International Organization for Standardization, Geneva, Switzerland. 2005.

[9] Huo H, Bobet A, Fernández G, Ramirez J. Analytical solution for deep rectangular structures subjected to far-field shear stresses. Tunnelling and Underground Space Technology. 2006; 21(6): 613–625.

[10] Bobet A, Fernandez G, Huo H, Ramirez J. A practical iterative procedure to estimate seismic-induced deformations of shallow rectangular structures. Canadian Geotechnical Journal. 2008; 45(7): 923–938.

[11] Anderson DG, Geoffrey RM, Ignatius L, Wang JN. Seismic analysis and design of retaining walls, buried structures, slopes and embankments. NCHRP Report 611, Transportation Research Board. 2008.

[12] FHWA (Federal Highway Administration). Technical manual for design and construction of road tunnels-civil elements. Department of transportation, Federal Highway Administration, Washington D.C., U.S 2009.

[13] Bobet A. Drained and undrained response of deep tunnels subjected to far-field shear loading. Tunnelling and Underground Space Technology. 2010; 25(1): 21–31.

[14] Ulgen D, Saglam S, Ozkan MY. Dynamic response of a flexible rectangular underground structure in sand: centrifuge modeling. Bulletin of Earthquake Engineering. 2015; 13(9): 2547–2566.

[15] Tsinidis G, Rovithis E, Pitilakis K, Chazelas JL. Seismic response of box-type tunnels in soft soil: experimental and numerical investigation. Tunnelling and Underground Space Technology. 2016; 59: 199–214.

[16] Shibayama S, Izawa J, Takahashi A, Takemura J, Kusakabe O. Observed behaviour of a tunnel in sand subjected to shear deformation in a centrifuge. Soils and Foundations. 2010; 50(2): 281–294.

[17] Chou JC, Kutter BL, Travasarou T, Chacko JM. Centrifuge modeling of seismically induced uplift for the BART transbay tube. Journal of Geotechnical and Geoenvironmental Engineering, 2011; 137(8): 754–765.

[18] Cilingir U, Madabhushi SPG. A model study on the effects of input motion on the seismic behaviour of tunnels. Soil Dynamics and Earthquake Engineering. 2011; 31(3): 452–462.

[19] Cilingir U, Madabhushi SPG. Effect of depth on the seismic response of square tunnels. Soils and Foundations, 2011; 51(3): 449–457.

[20] Chian SC, Madabhushi SPG. Effect of buried depth and diameter on uplift of underground structures in liquefied soils. Soil Dynamics and Earthquake Engineering. 2012; 41: 181–190.

[21] Lanzano G, Bilotta E, Russo G, Silvestri F, Madabhushi SPG. (2012). Centrifuge modeling of seismic loading on tunnels in sand. Geotechnical Testing Journal. 2012; 35(6): 1–15.

[22] Moss RES, Crosariol VA. Scale model shake table testing of an underground tunnel cross section in soft clay. Earthquake Spectra. 2013; 29(4): 1413–1440.

[23] Chen G, Wang Z, Zuo X, Du X, Gao H. Shaking table test on the seismic failure characteristics of a subway station structure on liquefiable ground. Earthquake Engineering and Structural Dynamics. 2013; 42(10): 1489–1507.

[24] Chen G, Chen S, Zuo X, Du X, Qi C, Wang Z. Shaking-table tests and numerical simulations on a subway structure in soft soil. Soil Dynamics and Earthquake Engineering. 2015; 76: 13–28.

[25] Tsinidis G, Pitilakis K, Madabhushi G, Heron C. Dynamic response of flexible square tunnels: centrifuge testing and validation of existing design methodologies. Géotechnique. 2015; 65(5): 401–417.

[26] Ulgen D, Saglam S, Ozkan MY. Assessment of racking deformation of rectangular underground structures by centrifuge tests. Géotechnique Letters. 2015; 5(4): 261–268.

[27] Hashash YMA, Park D, Yao JIC. Ovaling deformations of circular tunnels under seismic loading, an update on seismic design and analysis of underground structures. Tunnelling and Underground Space Technology. 2005; 20(5): 435–441.

[28] Anastasopoulos I, Gerolymos N, Drososi V, Kourkoulis R, Georgarakos T. Nonlinear response of deep immersed tunnel to strong seismic shaking. Journal of Geotechnical and Geoenvironmental Engineering, 2007; 133(9): 1067–1090.

[29] Anastasopoulos I, Gerolymos N, Drosos V, Georgarakos T, Kourkoulis R, Gazetas G. Behaviour of deep immersed tunnel under combined normal fault rupture deformation and subsequent seismic shaking. Bulletin of Earthquake Engineering. 2008; 6(2): 213–239.

[30] Amorosi A, Boldini D. Numerical modelling of the transverse dynamic behaviour of circular tunnels in clayey soils. Soil Dynamics and Earthquake Engineering. 2009; 29(6): 1059–1072.

[31] Kontoe S, Zdravkovic L, Potts DM, Menkiti CO. On the relative merits of simple and advanced constitutive models in dynamic analysis of tunnels. Géotechnique. 2011; 61(10): 815–829.

[32] Kontoe S, Avgerinos V, Potts DM. Numerical validation of analytical solutions and their use for equivalent-linear seismic analysis of circular tunnels. Soil Dynamics and Earthquake Engineering. 2014; 66: 206–219.

[33] Debiasi E, Gajo A, Zonta D. On the seismic response of shallow-buried rectangular structures. Tunnelling and Underground Space Technology. 2013; 38: 19–113.

[34] Baziar MH, Moghadam MR, Kim DS, Choo YW. Effect of underground tunnel on the ground surface acceleration. Tunnelling and Underground Space Technology. 2014; 44: 10–22.

[35] Lanzano G, Bilotta E, Russo G, Silvestri F. Experimental and numerical study on circular tunnels under seismic loading. European Journal of Environmental and Civil Engineering. 2015; 19(5): 539–563.

[36] Tsinidis G. Response characteristics of rectangular tunnels in soft soil subjected to transversal ground shaking. Tunnelling and Underground Space Technology. 2017; 62: 1–22.

[37] Bao X, Xia Z, Ye G, Fu Y, Su D. Numerical analysis on the seismic behavior of a large metro subway tunnel in liquefiable ground. Tunnelling and Underground Space Technology. 2017; 66: 91–106.

[38] Abate G, Massimino MR. Numerical modelling of the seismic response of a tunnel-soil-aboveground building system in Catania (Italy). Bulletin of Earthquake Engineering. 2017; 15(1): 469–491.

[39] Tinawi R, Sarrazin M, Filiatrault A. Influence of soft clays on the response spectra for structures in eastern Canada. Soil Dynamics and Earthquake Engineering. 1993; 12(8): 469–477.

[40] Pan TC. Site-dependent building response in Singapore to long-distance Sumatra earthquakes. Earthquake spectra. 1997; 13(3): 475–488.

[41] Mayoral JM, Alberto Y, Mendoza MJ, Romo MP. Seismic response of an urban bridge-support system in soft clay. Soil Dynamics and Earthquake Engineering. 2009; 29(5), 925–938.

[42] Banerjee S, Goh SH, Lee FH. Earthquake-induced bending moment in fixed-head piles in soft clay. Géotechnique. 2014; 64(6): 431–446.

[43] Goh SH, Zhang L. Estimation of peak acceleration and bending moment for pile-raft systems embedded in soft clay subjected to far-field seismic excitation. Journal of Geotechnical and Geoenvironmental Engineering. 2017; 143(11): 04017082.

[44] Banerjee S. Centrifuge and Numerical modeling of soft clay-pile-raft foundations subjected to seismic shaking. Ph.D. thesis, National University of Singapore. 2009.

[45] Zhang L. Centrifuge and numerical modelling of the seismic response of pile groups in soft clays. Ph.D. thesis, National University of Singapore. 2014.

[46] Zhang L, Goh SH, Yi J. A centrifuge study of the seismic response of pile–raft systems embedded in soft clay. Géotechnique, 2017; 67(6): 479–490.

[47] Dadashi D, Nasserasadi K. Seismic damages comparison of low-rise moderate reinforced concrete moment frames in the near- and far-field earthquakes by a probabilistic approach. Internation Journal of Advanced Structural Engineering. 2015; 7: 171–180.

[48] Bhandari M, Bharti SD, Shrimali MK, Datta TK. The numerical study of base-isolated buildings under near-field and far-field earthquakes. Journal of Earthquake Engineering. 2017; 1: 1–19.

[49] Lysmer J, Kuhlemeyer RL. Finite dynamic model for infinite media. Journal of the Engineering Mechanics Division. 1969; 95(4–6): 859–877.

[50] Semblat JF, Brioist JJ. Efficiency of higher order finite elements for the analysis of seismic wave propagation. Journal of Sound and Vibration. 2000; 231(2): 460–7.

[51] Zhang L, Goh SH, Liu H. Seismic response of pile-raft-clay system subjected to a long-duration earthquake: centrifuge test and finite element analysis. Soil Dynamics and Earthquake Engineering. 2017; 92: 488–502.

[52] Viggiani G, Atkinson JH. Stiffness of fine-grained soil at very small strains. Géotechnique. 1995; 45(2): 249–265.

[53] Zhang L, Liu H. Seismic response of clay-pile-raft-superstructure systems subjected to far-field ground motions. Soil Dynamics and Earthquake Engineering. 2017; 101: 209–224.

[54] Kramer SL. Geotechnical earthquake engineering. Prentice Hall, Upper Saddle River, N.J 1996.

[55] Zhang L, Liu Y, Hu J. Statistical analysis of earthquake-induced bending moment in fixed-head piles embedded in soft clay. Journal of Engineering Mechanics. 2017; 143(9): 04017059.

[56] Liu Y, Zhang L. Seismic response of pile-raft system embedded in spatially random clay. *Géotechnique*. 2018 10.1680/jgeot.17.T.015 (in press)

